# Polymeric Materials Based on Carbon Dioxide: A Brief Review of Studies Carried Out at the Faculty of Chemistry, Warsaw University of Technology

**DOI:** 10.3390/polym14040718

**Published:** 2022-02-13

**Authors:** Zbigniew Florjańczyk, Gabriel Rokicki, Paweł Grzegorz Parzuchowski, Magdalena Mazurek-Budzyńska, Maciej Dębowski

**Affiliations:** Faculty of Chemistry, Warsaw University of Technology, Noakowskiego 3, 00-664 Warsaw, Poland; gabro@ch.pw.edu.pl (G.R.); pparzuch@ch.pw.edu.pl (P.G.P.); mmazurek@ch.pw.edu.pl (M.M.-B.)

**Keywords:** CO_2_-based polymers, cyclic carbonates, carbon dioxide capture, hyperbranched polymers, non-isocyanate polyurethanes, poly(hydroxy-urethane)s, transurethanization

## Abstract

Carbon dioxide is an important raw material in many industrial technologies, but it is also one of the greenhouse gases that has to be effectively removed from the environment. This contribution provides a brief overview of carbon dioxide-based polymers developed in the laboratories of the Faculty of Chemistry at Warsaw University of Technology. We present some simple and versatile synthetic approaches that can be used to prepare a library of oligocarbonate diols, polycarbonates, poly(ester-carbonates), poly(ether-carbonates) and various types of polyurethanes, including the newly emerging family of environmentally friendly non-isocyanate polyurethanes. The main synthesis strategy involves the reaction of CO_2_ with oxiranes to form five-membered cyclic carbonates, which can be utilized as a source of carbonate bonds in polymeric materials obtained by the ester exchange reactions and/or step-growth polyaddition. We also show that cyclic carbonates are valuable starting materials in the synthesis of hyperbranched polymers and polymer networks. The properties of several CO_2_-based polymers are presented and their potential application as biomaterials, smart materials, and absorbers with a high CO_2_ capture capacity is discussed.

## 1. Introduction

The Faculty of Chemistry at the Warsaw University of Technology is one of the few Polish academic centers where the research and teaching activities in the field of polymeric materials began before World War II. The pioneer of this subject was professor Kazimierz Smoleński, Head of the Department of General Organic Technology and Carbohydrate Technology [1]. In 1936, his initiative research works were undertaken on the synthesis and polymerization of methyl methacrylate, which led to the development of a technology for the production of poly(methyl methacrylate) on a semi-technical scale. He also made a significant contribution to the development of the technology of synthetic butadiene rubber from ethanol, which was put into production in 1939 at the Dębica Plant by a team of employees of the Chemical Research Institute, led by a graduate of the Faculty of Chemistry, Eng. Wacław Szukiewicz [2]. After the death of Professor Smoleński in 1943, his work was continued by Eng. Stanisław Porejko. He compiled the first textbook and, during World War II, gave lectures on plastics technology in the underground. In 1948, he established the first laboratory for education and research in the field of plastics, located within the Faculty of Chemistry at the Warsaw University of Technology. In 1963, this laboratory obtained the status of a department, and in 2000, it was named the Chair of Chemistry and Technology of Polymers (CCTP).

The research topics carried out at the CCTP were very diverse. An important part of this was the development of new technologies, which were successively implemented in the industry. The most important achievements include the launch of the production of new types of varnish resins (Polifarb Dębica, 1988–1990), and a wide range of polymer dispersions used for the production of adhesives (Boryszew S.A., 1993–1995). A number of materials and products manufactured on a smaller scale, such as a new type of liquid hardener for epoxy resins and compositions for the sealing of optoelectronic devices, compositions for dental fillings, and systems for the irrigation of horticultural crops were also successfully implemented. In the last decade, the employees of the CCTP have made a significant contribution to the modernization of one of the stages of polypropylene production technology in the Basell Orlen Polyolefins plant located in Płock and to the construction of a model installation for the production of biodegradable lactide polymers on a scale of approximately 500 kg per year (as part of the BIOPOL and LACMAN projects, co-financed by the European Regional Development Funds).

**Scheme 1:**
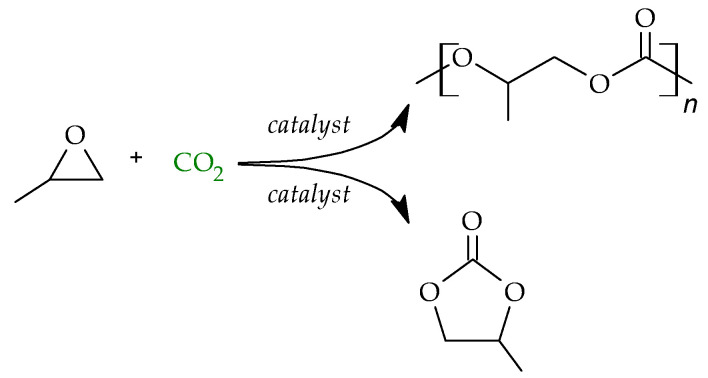
Products formed in the reaction of CO_2_ with propylene oxide.

At the same time, fundamental research works were carried out on the development of synthesis methods and the properties of many new polymeric materials. One of the leading topics in the scientific activity of the CCTP, continuously explored for the last 45 years, concerns the use of carbon dioxide in the synthesis of polymers. The early studies were stimulated by a breakthrough discovery in 1969 of the copolymerization of CO_2_ and propylene oxide, leading to the formation of high-molecular-weight poly(propylene carbonate) (Scheme 1) [3,4].

The original catalyst applied by Inoue et al. was a mixture of diethylzinc and water. This catalytic system was significantly improved in CCTP laboratories by Professor Witold Kuran and his students, who replaced water with organic compounds having two or more active hydrogens, especially trihydric phenols and dicarboxylic acids [5,6,7]. It should be noticed that Dr. Andrzej Rokicki, a student of Professor Kuran, continued this research in Air Products and Chemicals Inc. and was a member of the team that developed a very effective catalyst for the synthesis of poly(propylene carbonate)—this product of the reaction between zinc oxide and dicarboxylic acid was the first catalyst system used in industry [8]. Nowadays, for the copolymerization of CO_2_ with propylene oxide and other oxiranes, mainly mono- or bimetallic complexes with a well-defined structure are used, especially derivatives of Zn, Co, and Cr [9,10,11].

It should be noted that the thermodynamic products of the reaction between CO_2_ and epoxides are five-membered cyclic carbonates (1,3-dioxolan-2-ones) that are easily available in the presence of the appropriate catalysts. They cannot be applied as precursors for poly(alkene carbonates) due to the positive Gibbs free enthalpy of polymerization [12]; however, it has been found that some of them could undergo copolymerization with heterocyclic monomers [13,14]. Unfortunately, in most of the studied systems, the amount of carbonate units incorporated into the polymer chain was small. The only exception was the alternating copolymerization of ethylene carbonate with cyclic tetramethylene urea, carried out in the presence of dibutylmagnesium, which resulted in polyurethane (PUR) with an average molecular weight (M_n_) of ca. 20,000 [15]. From a practical point of view, the processes of crosslinking of epoxy resins in the presence of a cationic polymerization catalyst and five-membered cyclic carbonate as a modifier seem to be of particular interest. In these systems, a high degree of conversion of epoxy groups can be easily obtained, whereas the presence of a small amount of carbonate units improves the mechanical properties of the crosslinked resin [16].

Although the five-membered cyclic carbonates hardly undergo chain polymerization, they can be regarded as very promising reagents in the synthesis of many classes of condensation polymers and reactive resins. This approach will be illustrated by the results obtained in the CCTP and described in the following sections of this paper. We will also present the results of our studies on the application of linear dimethyl carbonate (DMC) in the synthesis of advanced polymeric materials, especially “non-isocyanate polyurethanes”, as well as our attempts to use polymeric matrices for carbon dioxide sequestration.

## 2. Synthesis of Five-Membered Cyclic Carbonates

**Scheme 2:**
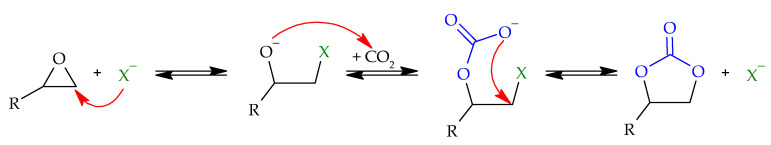
Mechanism of the five-membered cyclic carbonate synthesis from oxirane and CO_2_ catalyzed by alkali metal halide.

As already mentioned, the basic method for the synthesis of five-membered cyclic carbonates is the reaction of oxiranes with carbon dioxide. These reactions are carried out in the presence of catalysts, which are most often metal complexes, organic halides, or ion-exchange resins [13]. Our group developed a very effective catalytic system comprising crown ethers and alkali metal halides (e.g., KI), which enables, in many systems, the almost quantitative insertion of CO_2_ into an oxirane ring [13,17,18,19,20]. The catalytic effect of the metal halide in this reaction can be explained by the mechanism involving the halide anion attack on the α-carbon atom of the oxirane ring, resulting in the formation of the alcoholate ion—the latter captures the CO_2_ molecule and finally is transformed into a cyclic structure via an intramolecular nucleophilic substitution reaction (Scheme 2).

**Scheme 3:**
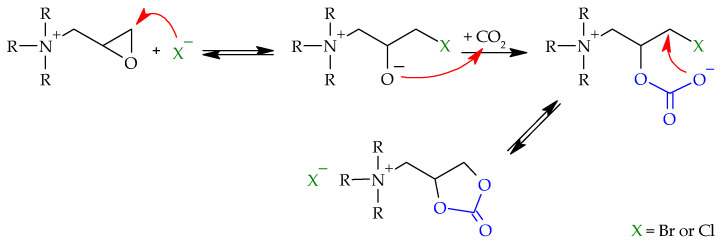
Mechanism of the CO_2_ fixing by oxiranes with a quaternary ammonium cationic center.

The presence of crown ether facilitates the solubilization of the salt and the formation of reactive “naked” anions. Quaternary ammonium halides are also frequently used as catalysts in these processes, but our observations show that systems with crown ethers are generally more effective. An interesting exception are oxirane derivatives with an ammonium cation at the β-position to the oxirane ring, which are able to fix carbon dioxide at room temperature and low CO_2_ pressure [13]. The reason for this high activity is probably the formation of zwitterions, which lower the energy of the transition states in this process (Scheme 3).

**Scheme 4:**
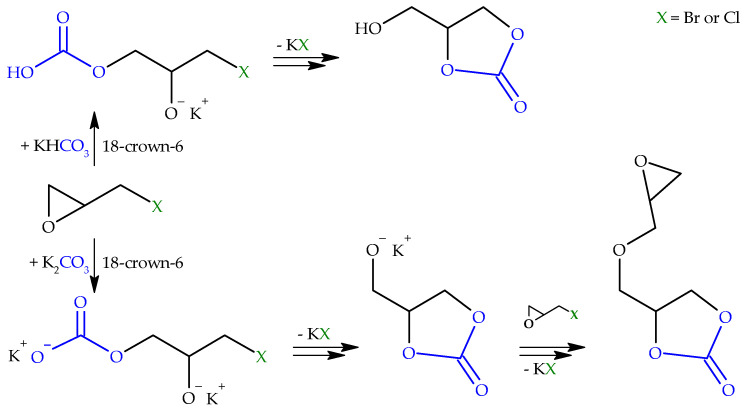
Synthesis of cyclic carbonates from epihalohydrins.

Two other methods of obtaining cyclic carbonates developed in the CCTP are based on reactions of epihalohydrin with potassium carbonate or potassium bicarbonate (Scheme 4), which are carried out in the presence of 18-crown-6 ether as a cation complexing agent [13]. These reactions yield functionalized cyclic carbonates containing in their structure an additional oxirane (3-glycidyloxypropylene carbonate) or hydroxyl (4-hydroxymethyl-1,3-dioxolan-2-one) group, which are valuable starting materials in the synthesis of branched polymers. We have also elaborated an alternative pathway leading to glycerol carbonate (4-hydroxymethyl-1,3-dioxolan-2-one), which is based on the reaction of DMC with glycerol, in the presence of potassium carbonate: a yield higher than 95% can be achieved with proper optimization of this process [20].

## 3. Oligocarbonate and Co-Oligocarbonate Diols as Semiproducts in the Synthesis of Various Polymeric Materials

**Scheme 5:**
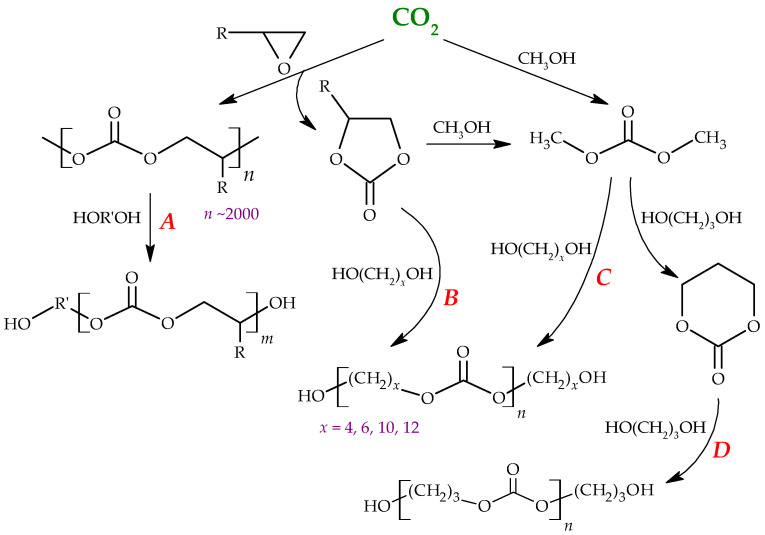
CO_2_ as a renewable starting material in the synthesis of oligocarbonate diols.

It was found that poly(carbonate-urethane)s obtained from oligocarbonate diols, unlike poly(ether-urethane)s or poly(ester-urethane)s, are resistant to hydrolysis and oxidation. Additionally, such polyurethanes exhibit also very good mechanical properties. As a source of carbonate linkages in the synthesis of oligocarbonate diols, one can use CO_2_ and its derivatives, such as phosgene, alkylene carbonates, and dialkyl or diphenyl carbonates. Taking into consideration the environmental impact, low price, availability of raw materials, and efficiency of the process, the CCTP focused its attention on the synthesis of oligocarbonate diols from simple carbonic acid esters (ethylene or propylene carbonate and DMC). In Scheme 5, four reaction pathways starting from CO_2_ as a renewable material, and leading to oligocarbonate diols with different numbers of carbon atoms in the repeating unit, are summarized. Oligocarbonate diols received according to reaction pathway *A* contain carbonate linkages separated by two carbon atoms. Oligomerols synthesized according to pathways *B* and *C* contain carbonate linkages separated by 4–12 carbon atoms, depending on the diol used.

The most promising method for obtaining oligocarbonate diols intended for use in the synthesis of PUR is based on reaction pathway *B*. Due to the cyclic structure of alkylene carbonate, an attack of hydroxyl groups of a diol can occur at both the carbonyl (ester exchange) and alkyl carbon atom (etherification and decarboxylation). CCTP research has shown that, depending on the used diol, catalyst, reaction temperature, and concentrations of the reagents, this process may proceed with a predominance of either etherification or ester exchange. In the case of a transesterification reaction between ethylene carbonate and α,ω-diols containing six or more methylene groups in a molecule, simple alkali metal salts, such as NaCl or NaBr, can be used as catalysts, as well as a reaction temperature lowered to 130–145 °C. This has significantly limited the formation of oxyethylene fragments [21]. The use of xylene or toluene as an azeotropic solvent of ethylene glycol resulted in the reduction of the co-distillation of cyclic carbonate and thus its losses [22]. It is worth noting that, because of the use of such physiologically inert catalysts, the resulting oligocarbonate diols can be readily applied in biomedicine. Moreover, titanium catalysts, such as Ti(OBu)_4_, are also very effective in the synthesis of oligocarbonate diols, especially when propylene carbonate is used [23]. In the CCTP, the synthesis of oligocarbonate diols from DMC as a source of carbonate linkages was also elaborated. It was found that by introducing into the reaction system a solvent with a boiling point between those of DMC (90 °C) and methanol (56 °C), one can prevent the loss of monomers during the process and easily control the molar mass of the resulting oligocarbonate diol [24].

### 3.1. Poly(ester-carbonate)s

DMC, when combined with dimethyl terephthalate (DMT) or poly(ethylene terephthalate) (PET) recyclate as the sources of aromatic–aliphatic ester linkages, can be also applied as a starting material in the production of poly(ester-carbonates). The resulting copolymer based on 1,4-butanediol and containing ca. 50 mol% of carbonate units exhibited better mechanical strength (37 MPa) than commercially available aliphatic–aromatic copolyester Ecoflex^®^, while keeping the thermal properties at the same level [25]. It should be noted that Ecoflex^®^ is based on an adipic acid ester instead of a carbonic acid one.

### 3.2. Poly(carbonate-urea-urethane)s

**Scheme 6:**
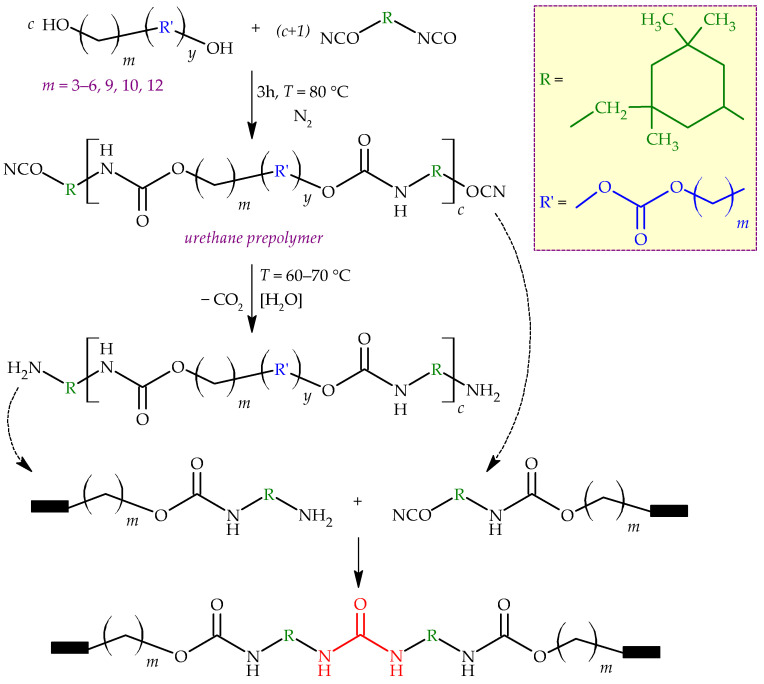
Synthesis of PCUU from oligo(alkylene carbonate) diol and diisocyanate.

The results of studies conducted in the CCTP indicate that oligo(alkylene carbonate)s can be applied as precursors of poly(carbonate-urea-urethane) (PCUU) soft segments. The syntheses is carried out without a solvent or catalyst, using aliphatic diisocyanate such as isophorone diisocyanate (IPDI) or hexamethylene diisocyanate (HMDI), with water vapor as a precursor of the chain extender (Scheme 6) [22,23]. The influence of the hydrocarbon chain length between the carbonate groups in oligo(alkylene carbonate)s on the final chemical structure, as well as the thermal and mechanical properties of poly(carbonate-urethane)s, was investigated [26]. It has been shown that products based on decamethylene and dodecamethylene oligocarbonates have lower crosslinking density (both covalent and physical) and higher content of the crystalline phase. Due to the presence of long hydrocarbon chains between their carbonate linkages, such polymers exhibit a higher elongation at break and lower glass transition temperature (T_g_). Moreover, some significant differences in the properties of the products related to the parity number of methylene groups between carbonate groups are also observed. For example, a characteristic minimum in the relationship between the T_g_ of the polymer and the number of CH_2_ groups in its repeating unit occurs in the case of PCUU derived from 1,9-nonanediol. Our studies indicate that urethane prepolymers containing lower concentrations of hydrocarbon fragments are also characterized by lower water vapor permeability, which leads to a much slower moisture-driven curing process. Because of this, the reaction of isocyanate groups with urethane and urea linkages proceeds to a higher extent and, as a consequence, the concentration of allophanate and biuret groups in such samples is higher.

As already mentioned, polycarbonate-based polyurethanes and polyureas are promising implant materials, especially for long-term implants, due to their improved resistance to hydrolytic degradation and lower tendency towards inflammation of the surrounding tissue in comparison to poly(ester-urethane)s. Therefore, the influence of the concentration of carbonate units within the soft segments on the hydrolytic stability of poly(carbonate-urea-urethane)s (PCUUs) in phosphate-buffered saline (PBS) solutions was also investigated in the CCTP [27]. The results of such studies, conducted over a period of 20 weeks, indicate that changes in the crystallinity, as well as the thermal and mechanical properties, of PCUUs strongly depend on the number of methylene units between the carbonate linkages in the soft segment of the polymer. These changes can be attributed to the thermal reorganization of the crystalline domains originating from these soft segments and occurring during prolonged annealing at 37 °C. Moreover, the annealed samples of PCUUs based on oligo(decamethylene carbonate) diol or oligo(dodecamethylene carbonate) diol exhibit an increase in Young’s modulus in comparison to samples not subjected to conditioning in PBS. It is assumed that, after the initial rupture of urea, these PCUUs regain their modulus through amine–isocyanate addition, resulting in the formation of new, less congested urea bonding.

### 3.3. Poly(carbonate-urea-urethane)s Showing a Shape-Memory Effect (SME)

Shape-memory polymers (SMPs) are classified as smart materials, which, under external stimuli, can change their shape between a permanent shape given by the processing method and a temporary shape given in the additional programming step. Thermoplastic shape-memory polyurethanes (SMPURs) are a special class of these materials, as they are well suited for a broad variety of processing methods. They are characterized by a phase-segregated structure, in which the switching (soft) domains act as temporary net points, and hard domains determine the permanent shape. However, in the case of such systems, the shape recovery rates are low due to stress relaxation and creep. In contrast, covalently crosslinked SMPs have shown high shape recovery ratios, but exhibit a limited capacity for elastic deformation. PCUUs elaborated in the CCTP have been found to exhibit very good shape-memory properties, manifesting both in the one-way shape-memory effect (1W-SME), as well as in the reversible bidirectional shape-memory effect (RB-SME). The first one requires the programming step after each deformation and recovery process, whereas shape changes in RB-SME can be reversibly changed many times, without external force or programming steps. The studies carried out in the CCTP show that highly crystallizable oligo(alkylene carbonate) diols with long hydrocarbon chains between carbonate linkages form temporary netpoints, whereas the permanent shape is defined by the small amount of hydrogen bonds, as well as covalent linkages within their hard segments (allophanate and biuret groups). Two types of isocyanate were used: IPDI [28] and lysine diisocyanate (LDI) [29]. The PCUU networks exhibited 1W-SME with programmed strains up to 1000%, whereby they provided excellent shape fixity (92–97% and 99% in the case of IPDI- and LDI-based PCUUs, respectively) and shape recovery (≥99%) ratios. The switching temperature varied within 36–65 °C for the IPDI-based PCUUs or 50–56 °C for the LDI-based PCUUs, and increased with the increasing molar mass of the oligo(alkylene carbonate) diol and the length of the hydrocarbon chain between the carbonate linkages.

Very recently, our SME PCUUs were utilized in the synthesis of multifunctional 4D actuators processable by 3D printing, and, after a proper programming step, they have shown shape changes in the stress-free condition [30]. When programmed with a strain of 1400%, the PCUU networks exhibited reversible elongation of up to 22%, and in the form of a gripper – reversible bending in the range of 37% to 60%, when the actuation temperature was varied between 45 °C and 49 °C.

### 3.4. Poly(urea-urethane)s Derived from Oligo(ester-carbonate) Diols (PECUUs)

Our group in the CCTP has also focused on the synthesis of poly(urea-urethane) capable of biodegradation, which is a crucial feature for its potential usage in the medical engineering field, especially for the production of resorbable sutures or implants, as well as systems for controlled drug release. Oligocarbonates, in contrast to oligoesters, degrade without the formation of carboxylic acids—only CO_2_ and diols are produced. In the case of using aliphatic polyesters as biomaterials, a high local concentration of carboxylic acids leads to the harmful acidification of organisms [31]. Moreover, the hydrolysis of the carbonate linkage is accompanied by a much lower inflammatory reaction in the tissues surrounding the implemented material [32].

We have designed a series of aliphatic oligo(ester-carbonate)s exhibiting a reduced ability to crystallize [33]. Low content of the crystalline phase facilitates the penetration of enzymes and accelerates the biodegradation processes. The results of our experiments on the hydrolytic and enzymatic degradation of poly(ester-carbonate-urea-urethane)s containing derivatives of succinic or adipic acid prove that by adjusting the molar ratio of ester and carbonate units in the flexible segment of PUR, the time of degradation can be easily controlled. For example, biodegradation tests were conducted on the amorphous PECUUs derived from asymmetrical IPDI and oligo(tetramethylene carbonate-*co*-succinate)s containing 57–79 mol% carbonate units in the soft segments. Three different solutions reported in the literature [34,35,36] as actively degrading ester and urethane groups were used: phosphate buffer (pH = 7.28), phosphate buffer with *Pseudomonas cepacia* lipase, and phosphate buffer with *Pseudomonas* sp. lipase. Our results indicated that only the samples containing less than 65 mol% carbonate units in the soft segment of PUR were prone to enzymatic and hydrolytic degradation, with the enzyme of *Pseudomonas* sp. exhibiting the highest degradation efficiency (the effects of the *Pseudomonas cepacia* enzyme or simple hydrolytic degradation were on a comparable level) [33]. In another study, we have shown that PECUUs based on adipic acid derivatives are characterized by a lower glass transition temperature, higher elongation at break, and higher tensile strength compared to PECUUs containing succinate groups. Analysis of water sorption and changes in the pH of the solution after immersion tests indicates that the samples most prone to water penetration and hydrolysis were those obtained from IPDI [37]. Furthermore, the resistance of these PECUUs to biological colonization, i.e., against *Candida* species, as well as their hemocompatibility and lack of cytotoxicity were examined. Our results indicated that IPDI-derived PECUUs exhibit anti-biofilm properties, which make them useful materials for potential medical applications, e.g., as implant materials in regenerative medicine [37]. Since the hemolysis of the examined samples was in the range of 3–10%, they were characterized by high but variable hemocompatibility. It is worth noting that these PECUUs are also able to support the growth of human keratinocytes (HaCaT) on their surfaces when coated with collagen [37].

PECUUs obtained in the CCTP can be also utilized as components in blends with polysulfone, producing partly degradable hollow-fiber membranes [38,39,40]. The conducted tests clearly showed that parts of the membranes related to PECUUs are partially degraded while maintaining a constant membrane cut-off point.

The Rokicki group worked as well on the design and synthesis of carbonate-based aliphatic–aromatic soft segments of polyurethanes. As the aromatic unit precursors, terephthalic acid esters (e.g., DMT) or the products of the alcoholysis of waste PET bottles with α,ω-diols can be used. The introduced aromatic units are responsible for an increase in the thermal stability of PECUUs compared to PCUUs, as well as significant improvements in mechanical properties—one can easily achieve tensile strength up to 60 MPa and elongation at break of 320%. It is worth noting that the additional incorporation of isophthalate units leads to polymeric products that still exhibit the amorphous structure, even if the content of aromatic units in the soft segments of PECUUs is as high as 53 mol% [41].

### 3.5. Poly(carbonate-siloxane-urethane)s

Poly(carbonate-urethane)s, due to their high resistance to oxidation and hydrolytic agents, are very attractive materials for the production of adhesives and coatings with increased resistance to weather conditions, as well as biomaterials. Taking the above into account, in our laboratories, polyurethanes based on oligo(carbonate-dimethylsiloxane) diols have been developed. Due to the potential antithrombogenic properties, such polymers are specifically dedicated to the production of biomaterials that come into contact with blood [42].

## 4. Non-Isocyanate Polyurethanes (NIPUs)

Diisocyanates used in the synthesis of classical polyurethanes raise severe health hazard concerns. Therefore, there is a growing demand for environmentally friendly processes and monomers.

Non-isocyanate polyurethanes (NIPUs) can be synthesized via two main routes:

- polycondensation, e.g., transurethanization;
- step-growth polyaddition, e.g., synthesis of poly(hydroxy-urethanes) (PHUs).

### 4.1. Non-Isocyanate Polyurethanes Obtained via Transurethanization

**Scheme 7:**
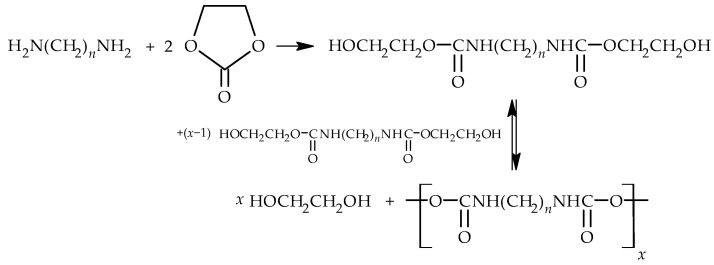
Synthesis of PUR from diamine and ethylene carbonate.

In the 1950s, Dyer and Scott used alkylene bis(2-hydroxyethylcarbamate)s as precursors for the synthesis of PUR without the utilization of phosgene and isocyanates (Scheme 7) [43].

**Scheme 8:**
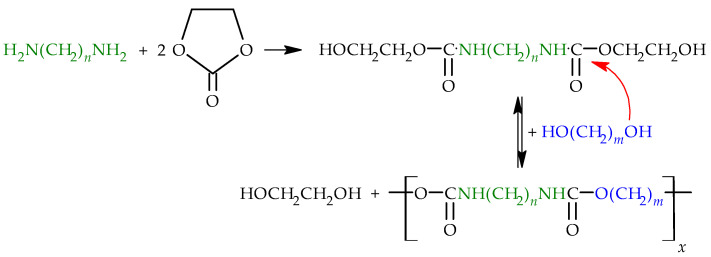
Synthesis of NIPU from α,ω-diamines, α,ω-diols, and ethylene carbonate.

In the CCTP, another kind of polycondensation method for NIPU synthesis was proposed that utilized aliphatic α,ω-diamines, α,ω-diols, and ethylene carbonate as monomers [44]. Firstly, in the reaction of diamine with ethylene carbonate alkylene, bis(2-hydroxyethylcarbamate)s are formed, which subsequently undergo a transurethanization reaction with α,ω-diol (Scheme 8). The whole process is carried out at 145–150 °C in the presence of a tin catalyst (e.g., dibutyltin oxide, Bu_2_SnO), resulting in [*n*,*m*]-polyurethane and a side product, ethylene glycol, which is readily removed from the reaction mixture under a vacuum.

**Scheme 9:**
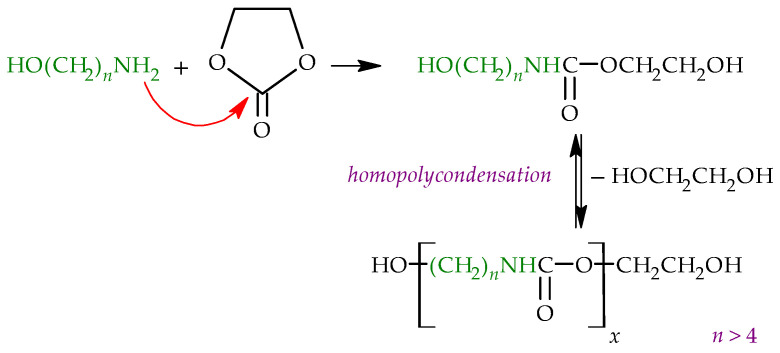
Synthesis of NIPU from α,ω-aminoalcohol and ethylene carbonate.

Alternatively, α,ω-aminoalcohol containing four and more CH_2_ groups in a molecule can be utilized instead of diamine and diol, leading to the formation of [*n*]-polyurethane as the transurethanization product (Scheme 9) [44]. It is worth noting that, in addition to urethane linkages, there is a small quantity of urea linkages present in the backbone of the obtained polymer.

Very recently, it has been found that if oligocarbonate diols are used instead of “short” diols, a high-molecular-weight PUR with only traces of urea linkages can be obtained [45]. Such a polymer has a very similar chemical structure to that obtained according to a conventional isocyanate pathway (e.g., from hexamethylene diisocyanate) and exhibits excellent mechanical properties—tensile strength higher than 40 MPa and elongation at break of up to 600%.

### 4.2. Non-Isocyanate Poly(hydroxy-urethane)s Obtained from Bis- and Multi(cyclic carbonate)s

**Scheme 10:**
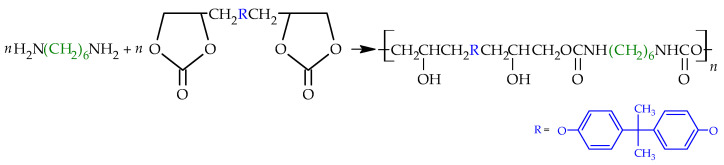
Non-isocyanate PHU synthesis from diamine and bis(cyclic carbonate).

The second most important pathway to NIPUs is based on the synthesis of PHUs in the reaction of bis- and multi(cyclic carbonate)s with aliphatic di- and polyamines (Scheme 10).

The step-growth polyaddition of five-membered bis(cyclic carbonate)s and diamines was reported as early as 1957 [46]. The resultant PHUs contained pendant primary and secondary hydroxyl groups. These hydroxyl groups are capable of forming intermolecular and intramolecular hydrogen bonds with urethane carbonyl groups. The intramolecular hydrogen bonds protect the carbonyl carbon atom, resulting in a decrease in the susceptibility of urethane linkages to hydrolysis.

The same approach was applied for the modification of epoxy resins cured with aliphatic polyamines [17,47]. It should be mentioned that this bis(cyclic carbonate) can be also used for obtaining high-molecular-weight poly(hydroxy-ethers) via its reaction with various bisphenols; in contrast to a typical method based on the diglycidyl ether of bisphenol A, in this new synthetic pathway, side reactions are greatly suppressed [48].

Very recently, PHUs have been applied as polymeric materials, showing energy-dissipating properties. The materials were obtained by the ring-opening polyaddition reaction of hyperbranched multi(cyclic carbonate) with various diamines [49].

**Scheme 11:**
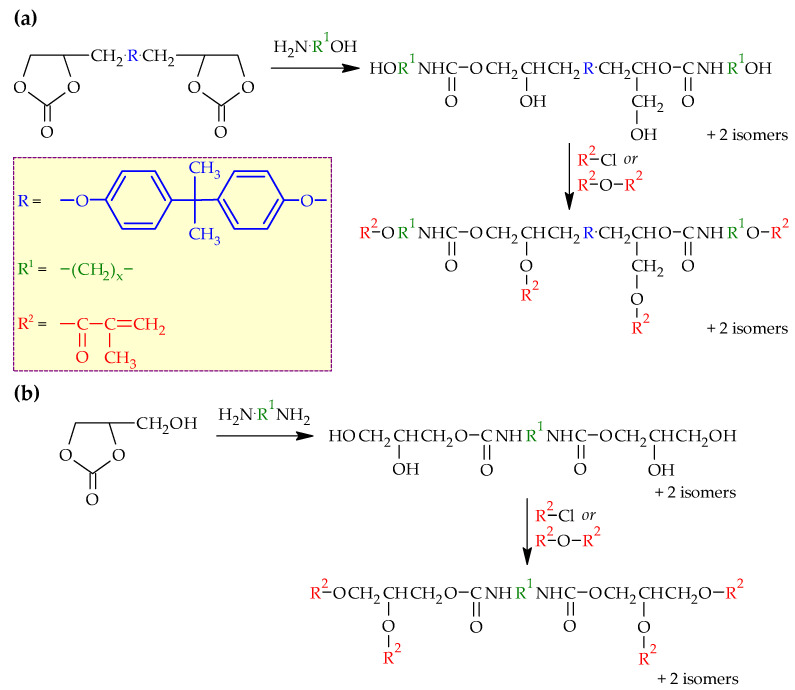
Non-isocyanate routes to photopolymerizable multimethacrylic monomers with urethane linkages, based on such starting materials as (**a**) bis(cyclic carbonate) and α,ω-aminoalcohol; (**b**) glycidol and diamine.

The non-isocyanate route can be also applied in the synthesis of photopolymerizable multimethacrylic monomers containing urethane linkages (Scheme 11). This type of resin exhibits very low polymerization shrinkage (ca. 3%) and low oxygen inhibition [50]. The mechanical properties of dental restoration materials based on such multimethacrylic monomers are also excellent (e.g., flexural strength more than 100 MPa and microhardness of 65 kPa) [51].

### 4.3. Poly(hydroxy-urethane)s Based on Five- and Six-Membered Bis(cyclic carbonate)s

**Scheme 12:**
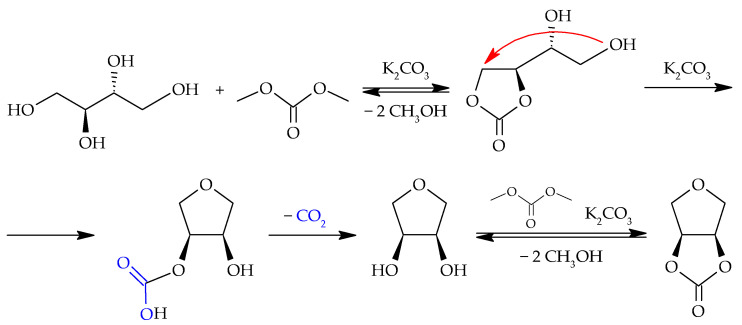
Synthesis of (3*R*,4*S*)-3,4-dihydroxytetrahydrofuran and (1*R*,5*S*)-2,4,7-trioxa-3-oxy-bicyclo[3.3.0]octane from *meso*-erythritol and DMC.

**Scheme 13:**
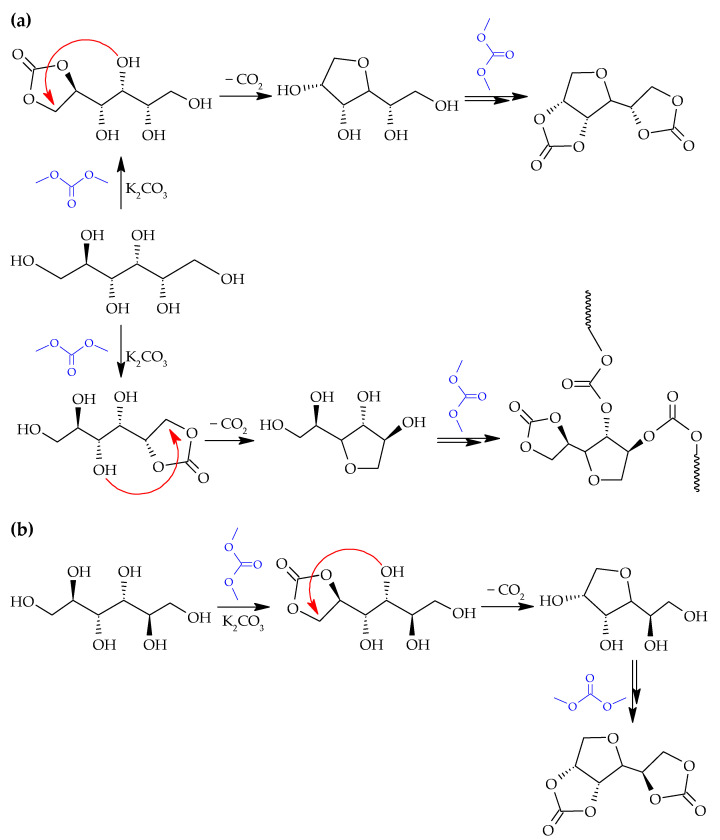
Reaction pathways to bis(cyclic carbonate)s, starting from DMC and (**a**) D-sorbitol; (**b**) D-mannitol.

A one-pot synthetic route to tetrahydrofuran derivatives, which were unexpectedly produced under basic conditions by the intramolecular etherification of substituted five-membered cyclic carbonates, was developed in the CCTP [52]. When *meso*-erythritol containing four vicinal hydroxyl groups is reacted with DMC at the stoichiometric ratio at 90 °C, (3*R*,4*S*)-3,4-dihydroxytetrahydrofuran is obtained with 86% yield. However, when the reaction is carried out with molar excess of DMC under the same reaction conditions, a bicyclic derivative of tetrahydrofuran and 1,3-dioxolan-2-one forms almost quantitatively (yield 96%) (Scheme 12). Furthermore, by replacing erythritol with D-mannitol or D-sorbitol, one can synthesize in this process bis(cyclic carbonate)s (Scheme 13).

**Scheme 14:**
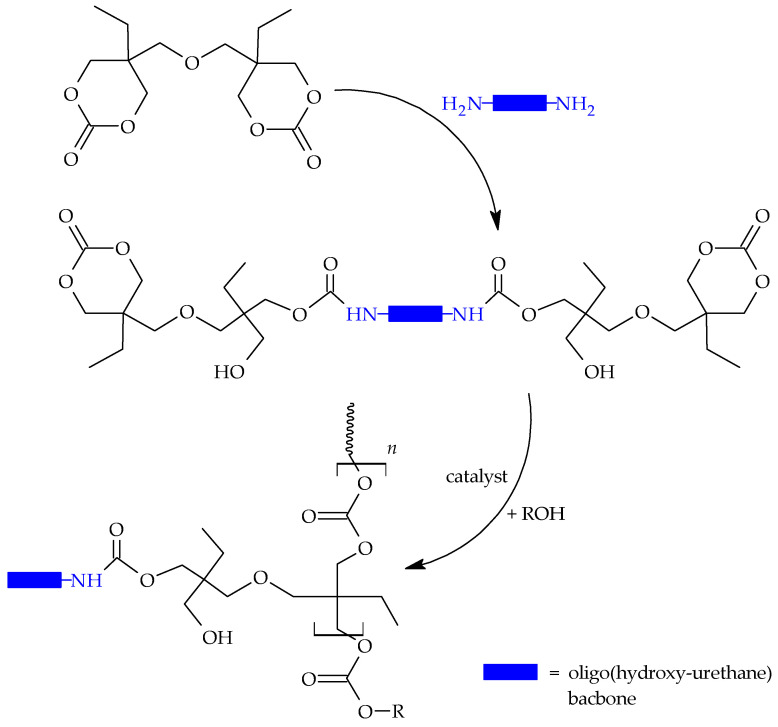
Synthesis of poly(carbonate-urethane) thermosets.

Bis(cyclic carbonate)s obtained from D-sorbitol or D-mannitol can be reacted with aliphatic diamines, resulting in the amine-terminated oligomeric PHUs. The latter, in combination with a derivative of di(trimethylolpropane) bearing six-membered carbonate rings, can be polymerized into a wide variety of crosslinked poly(carbonate-urethane)s (Scheme 14). Depending on the type of aliphatic diamine and the molar ratio of five- to six-membered bis(cyclic carbonate)s, the obtained NIPUs exhibit elastomeric or rigid properties [53].

It is worth mentioning that the reaction of diamine with cyclic carbonate (e.g., ethylene carbonate) leads to diols, which can be used to obtain polyurethanes according to classical procedures, e.g., in the reaction with di- or polyisocyanates [54,55]. Similar diols, α,ω-bis(2-hydroxyethoxy-carbonyl-amino)alkanes obtained from ethylene carbonate and diamines, were used by the Rokicki group as initiators for the polymerization of six-membered cyclic carbonates (e.g., trimethylene carbonate) to afford oligocarbonate diols (Scheme 5, path *D*) [56].

## 5. Hyperbranched Polymers (HBPs)

Dendrimers are highly branched molecules with repeating units emanating from a central core in a regular three-dimensional structure. Due to their unique properties, they are attractive for applications in many fields, including biology and materials sciences, as well as catalysis. However, their synthesis often involves multiple tedious steps of protection/deprotection and complicated purification.

In contrast to dendrimers, HBPs can be prepared according to one synthetic step only. Although HBPs contain linear units as insufficient branching, they still inherit the properties of dendrimers, such as good solubility, low viscosity, and multi-functionality at end groups.

### 5.1. Hyperbranched Polyethers from Renewable Resources

The research on HPBs conducted in the CCTP yielded several new structures based on renewable resources, such as carbon dioxide and glycerol.

**Scheme 15:**
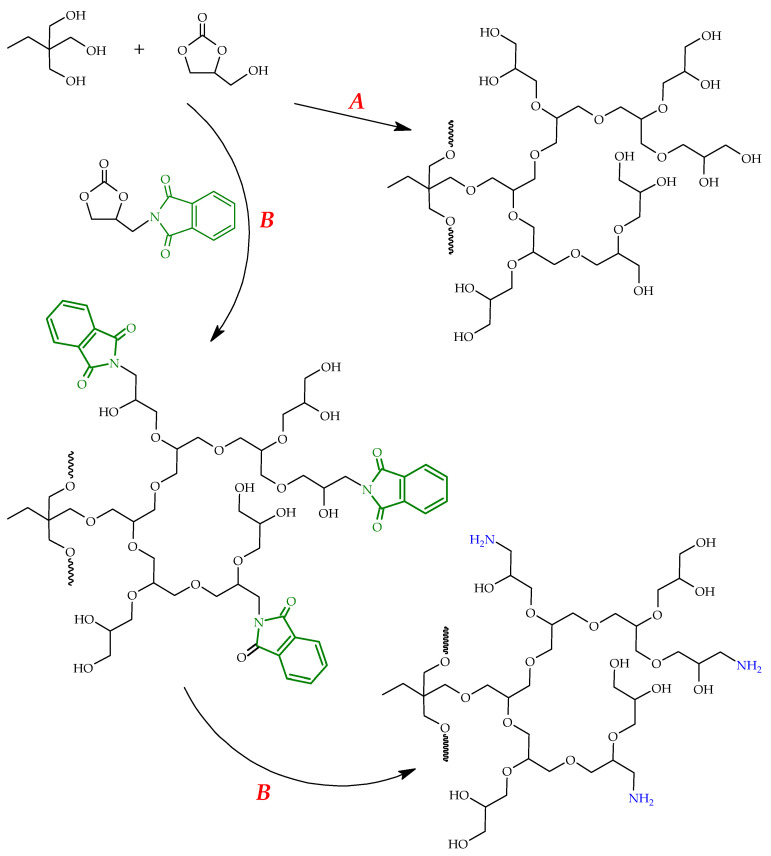
Synthesis of hyperbranched polyglycerols (path *A*) and aminated polyglycerols (path *B*) using cyclic carbonate monomers.

A hyperbranched aliphatic polyether with hydroxyl end groups can be produced from glycerol carbonate, an environmentally benign monomer obtained from renewable starting materials: glycerol and dimethyl carbonate (Scheme 15, path *A*) [20]. The anionic polymerization of glycerol carbonate, which proceeds with simultaneous decarboxylation, is performed using partially deprotonated trimethylolpropane (TMP) as an initiator. Pendant hydroxyl groups facilitate the ring-opening, multibranching polymerization, leading to a hyperbranched polyether. ^13^C NMR analysis of the polymerization products confirmed the presence of linear, dendritic, and terminal repeating units. MALDI-TOF mass spectrum analysis confirmed the presence of TMP and a glycerol core containing branched structures, as well as a relatively small amount of macromolecules with cyclic groups. It worth noting that such polymers are soluble in water, THF, methanol, and DMSO.

Glycerol carbonate undergoes also copolymerization with cyclic carbonate-bearing phthalimide groups, namely *N*-[(2-oxo-1,3-dioxolan-4-yl)methyl]phthalimide (Scheme 15, path *B*). The obtained polymer, after hydrazinolysis of phthalimide moieties, yields hyperbranched aliphatic polyethers containing hydroxyl and amine end groups [57]. During such a process, an intermediate, glycidol, is formed and acts as an initiator or monomer.

It should be mentioned that monomers with a more complex structure, such as those containing two cyclic carbonate moieties attached to a 4-hydroxyaniline core, were also studied in the CCTP. A great example of this is *N*,*N*-bis(2,3-dihydroxypropyl)-4-hydroxyaniline, which undergoes polycondensation with or without the starter (either trimethylolopropane triglycidyl ether or 2,2-bis[4-(2,3-dihydroxypropoxy)phenyl]propane decarbonate) to yield hyperbranched poly(hydroxyethers) [58].

**Figure 1:**
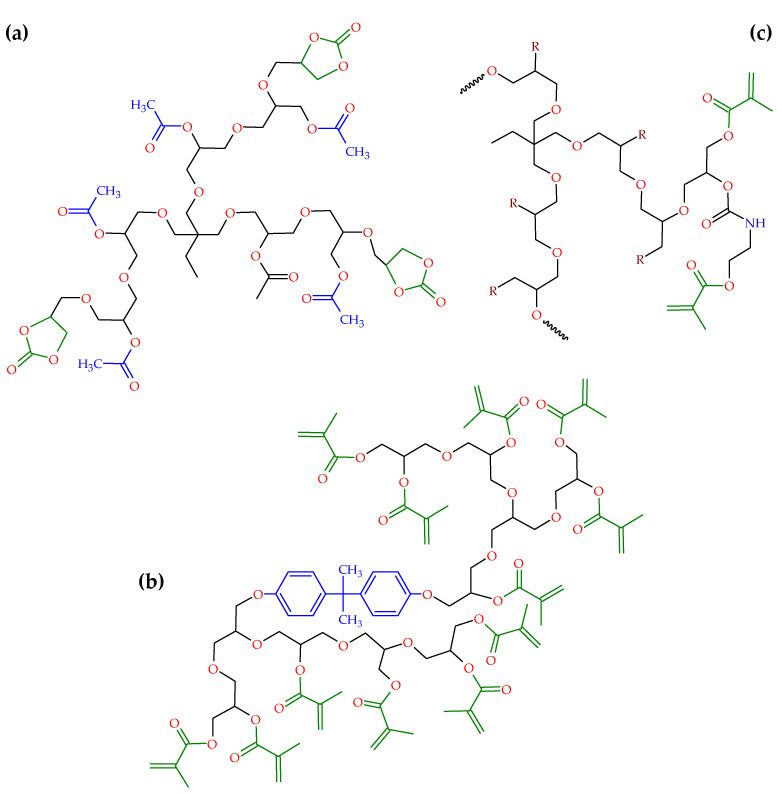
Functional hyperbranched polyglycerols containing: (**a**) cyclic carbonate and acetate groups, (**b**) methacrylate and urethane bonds and (**c**) methacrylate groups with bisphenol A core.

Hyperbranched polyglycerols (HBPGs) are valuable starting materials for the synthesis of more complex molecular systems, which can be used to modify the mechanical properties of epoxy resins. The main disadvantage associated with the application of these highly crosslinked thermosetting polymers is related to their inherent brittleness, which increases with crosslink density. The studies carried out in the CCTP indicate that terminal, vicinal hydroxyl groups of HBPGs can be easily converted in the presence of K_2_CO_3_ in the reaction with DMC into terminal five-membered cyclic carbonate groups [47]. The remaining OH groups of the polymer are easily protected via esterification with acetic anhydride to reduce the polymer’s hydrophilicity and increase its miscibility with the epoxy resin (Figure 1a), whereas a strong decrease in viscosity (from 73 to 3.6 Pa∙s) is also observed. The product of such a reaction can be used as a reactive toughness modifier for epoxy resins. The optimal mechanical properties, far surpassing those of the unmodified epoxy resin, are obtained for the compositions showing phase separation during the curing process.

Another interesting application of HBPGs containing five-membered cyclic carbonate moieties is the preparation and characterization of crosslinked non-isocyanate poly(hydroxyurethane) polymeric materials showing energy-dissipating properties [46]. These materials can be obtained by the ring-opening polyaddition reaction of hyperbranched multi(cyclic carbonate) with various diamines. Force absorbing efficiency tests show that these elastomers have the capability to dissipate around 60% of the energy and, in doing so, they are comparable to the shear thickening fluid materials that are currently used in human body protectors, vibration damping devices, or sound insulations. Thus, our studies prove that it is possible to obtain advanced engineering materials using environmentally friendly, glycerol- and CO_2_-based materials.

The hyperbranched polyethers can be also converted into new, low-viscosity and low-oxygen-inhibition hyperbranched multimethacrylate macromonomers capable of photopolymerization, for application in dental composites [59]. Two types of multimethacrylate resins have been studied in the CCTP—one based on a bisphenol A core and polyether branches (Figure 1b), and the other one based on TMP and containing urethane bonds (Figure 1c). The macromonomers were characterized by spectroscopic methods and the conversion of double bonds was investigated by FTIR. The physical properties of the multimethacrylate resins, such as viscosity, density, and polymerization shrinkage, were determined as well. The obtained results showed that such resins are attractive materials to use in composites, especially dental ones.

### 5.2. Hyperbranched Polyesters and Polycarbonates

Recent works conducted in the CCTP have explored the potential of six-membered cyclic carbonates containing free hydroxyl groups as precursors of hyperbranched polycarbonates. The obtained results indicate that, for 5-hydroxy-1,3-dioxan-2-one and its biscyclic ester derivative, no ring-opening polymerization can be observed; instead, isomerization to appropriate five-membered cyclic carbonates proceeds. The latter process occurs even if the hydroxyl groups are protected with an ester-type substituent [60]. However, in the case of other monomers, such as 5-[3-[(2-hydroxyethyl)thio]propoxy]-1,3-dioxan-2-one [61] and 5-(4-hydroxybutyl)-1,3-dioxan-2-one [62], the preparation of hyperbranched aliphatic polycarbonates is possible. Ring-opening polymerization of these monomers results in polymers containing solely primary hydroxyl groups. The number of linear, branching, and terminal units in these structures strongly depends on the catalyst used for polymerization. It is worth noting that linear analogues of polycarbonates can be prepared, too.

The idea of the preparation of glycerol-based hyperbranched polymers containing hydroxyl groups of equal reactivity can be further expanded to polyesters. For example, the self-condensing ring-opening polymerization of a simple lactone, 5-hydroxymethyl-1,4-dioxan-2-one (5-HDON), produces hyperbranched polymers, in which the repeating unit consists of glycerol and glycolic acid residues, thus making them potentially biodegradable and biocompatible [63]. The same reactivity of the primary OH groups enables a higher branching degree of the resulting polyester. Furthermore, 5-HDON can be a useful branching monomer for the ring-opening polymerization of other cyclic compounds [64]. For example, a glycerol-based hyperbranched polymer containing primary OH groups is produced via polycondensation of ethyl[3-[2-hydroxy-1-(hydroxymethyl)ethoxy]propylthioacetate over a wide range of catalysts and reaction conditions [65]. The degree of branching of the resulting polymers is in the range of 0.50–0.03, and they are susceptible to hydrolysis or alcoholysis under mild conditions.

### 5.3. Solubility of HBP in Supercritical Carbon Dioxide (scCO_2_)

Over the past 40 years, there has been intense interest in the use of scCO_2_ as an environmentally friendly solvent for laboratory and industrial applications. Its popularity stems from the fact that it is nontoxic, nonflammable, readily available in vast amounts, and that it is the second-least-expensive solvent after water. scCO_2_ is widely believed to be a good choice for a versatile solvent for polymer synthesis and processing. Unfortunately, carbon dioxide’s solvent power is low, especially for polar and high-molecular-weight polymers. The design and synthesis of the CO_2_-soluble surfactants, ligands, and phase transfer agents demand broad knowledge of the solubility of polymers in dense CO_2_.

Identification of highly CO_2_-soluble polymers has been a subject of intense research in the CCTP, especially in the case of hyperbranched polyesters and polyethers [66]. Moreover, the solubilities of modified hyperbranched polymers in light hydrocarbons have been also measured. The influence of the nature and number of end group functionalities, molecular weight, chemical structure of the interior of macromolecules, and the concentration of a supercritical gas on the phase behavior have been also studied. Parent HBPs bearing free hydroxyl groups hardly dissolve in scCO_2_, and the terminal OH groups have to be modified with a proper protecting agent, e.g., aliphatic carboxylic acid derivatives, trimethylchlorosilane, or trifluoroacetic acid anhydride. Modified polyesters show good solubility in scCO_2_, ethane, and propane. Our works and literature data show that the silane and fluorinated derivatives of hyperbranched polyesters are soluble in scCO_2_ to the same extent as the most CO_2_-philic polymeric materials synthesized till now. The nature of the end groups is an important factor that influences the phase equilibria of hyperbranched macromolecules in supercritical solvents. On the other hand, the nature of the interior of the macromolecule plays a significant role, too. Our results show that, in general, hyperbranched polyesters are more soluble in carbon dioxide than hyperbranched polyethers(polyglycerols), whereas, in light hydrocarbons applied as solvents, the opposite effect is observed.

The phase behavior of fluorinated hyperbranched polycarbonates in scCO_2_ was also explored as a function of concentration and temperature. The results of our works indicate that hyperbranched polycarbonates are reasonably soluble in scCO_2_, and the hyperbranched structure of a polymer in comparison to the linear one facilitates solubility even though the carbonate structural units do not promote solubility in scCO_2_ [62].

In addition, we performed some comprehensive studies on the influence of the structure of hyperbranched polyesters on their solubility in scCO_2_. Random hyperbranched copolyesters of 2,2-bis(hydroxymethyl)propionic acid (bis-MPA) with ε-caprolactone (ε-CL) and the products of their modification with fluorinated anhydrides or chlorotrimethylsilane have been thoroughly investigated. The obtained results showed that the content of chain extender units of 6-hydroxyhexanoic acid (formed from ε-CL) and terminal group functionalities play a key role in the solubility of the polymers in scCO_2_. The introduction of only small amounts (5−10%) of chain extender units into the dense structure of branched poly(bis-MPA) facilitates the solubility of this polymer in scCO_2_ [67].

**Figure 2:**
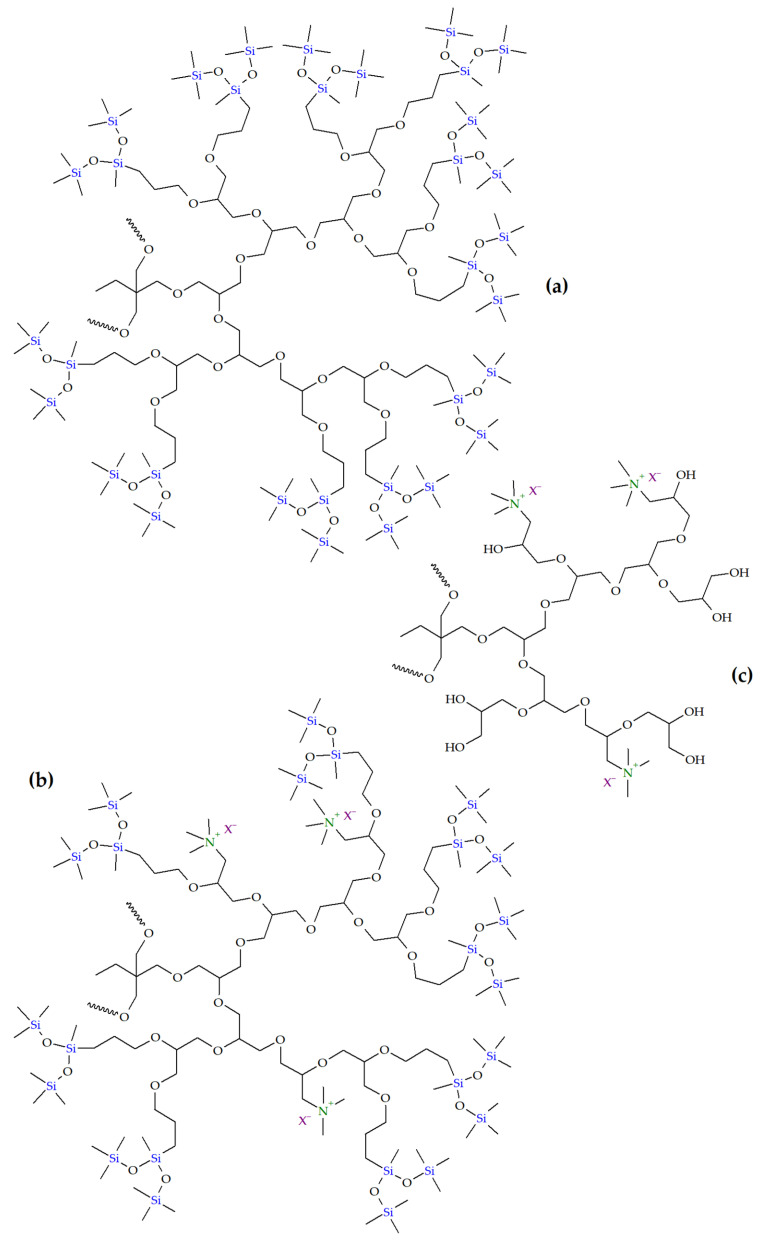
Modified hyperbranched polyglycerols developed in CCTP: (**a**) poly(ether-siloxane), (**b**) poly(ether-siloxane) with ammonium groups, (**c**) polyglycerol with ammonium groups.

The most spectacular scCO_2_ solubility results obtained in the CCTP concern hyperbranched poly(ether-siloxane)s. Such types of polymers (depicted in Figure 2a) exhibit very good solubility despite the fact that they contain hydrophilic polyether structures. Thus far, the incorporation of any type of substituent into the poly(dimethylsiloxane) (PDMS) chains results in a decrease in the solubility in scCO_2_. We showed that it is possible to obtain copolymers containing both hydrophilic (polyether) and hydrophobic (polysiloxane) parts, which have better solubility in scCO_2_ than the respective homopolymers [68].

The elaborated synthetic pathway allowed us to design a new polymeric catalyst for the addition of carbon dioxide to the oxirane ring, namely hyperbranched polyglycerols containing trimethylammonium groups and siloxane (Figure 2b) or hydroxyl end groups (Figure 2c) [69]. These siloxane derivatives can be synthesized with high yields in a five-step procedure including the anionic ring-opening copolymerization of glycidol with the phthalimide-epoxy monomer, followed by reactions with allyl bromide, hydrosililation with hydrogenheptamethyltrisiloxane, hydrazinolysis of phthalimide groups, and quaternization of the resulting amine groups with methyl iodide. Hydroxyl derivatives are obtained by the quaternization of previously reported aminated HBPGs with methyl iodide [57,70]. Both types of polymers are effective in catalysis with the addition of CO_2_ to oxirane. It is worth noting that the hydrophilic catalysts show higher efficiency, but the synthesis of ethylene carbonate is accompanied by the formation of small amounts of ethylene glycol. The siloxane-containing catalyst can be easily separated from the reaction mixture and presents strong potential in the process of converting CO_2_ into valuable chemical raw materials.

## 6. Reversible Carbon Dioxide Capture

Carbon dioxide is an important greenhouse gas that is released through natural processes such as respiration and volcanic eruptions, as well as human activities such as deforestation and burning fossil fuels. The CO_2_ concentration in the air is at its highest level in the last 650 thousand years, reaching level of 415 ppm (data on the monthly mean CO_2_ concentration measured in November 2021 at Mauna Loa Observatory, Hawaii) [71]. The climate catastrophe owing to the surplus of CO_2_ in the atmosphere is an immediate threat to our security and prosperity. Therefore, considerable effort has been focused on developing a range of chemical and physical methods for efficient CO_2_ capture and sequestration. Several review articles dealing with CO_2_ capture, specifically over the general solid adsorbents [72], nanoporous materials [73], metal–organic frameworks (MOFs) [74], ionic liquids [75], and membranes [76], have appeared in the literature.

One type of possible CO_2_ sorbent includes modified hyperbranched polyglycerols. Their hydroxyl groups not only increase the hydrophilicity, but also allow for their modification, leading to polymers with carboxyl, amine, or vinyl groups, as well as to polymers with bonded aliphatic and perfluorated chains.

In our works, we reported an easy synthetic pathway towards hyperbranched polyglycerols containing primary amine groups (A-HBPGs) [70]. A-HBPGs can be synthesized in a two-step procedure including the anionic ring-opening copolymerization of glycidol with the phthalimide-epoxy monomer, followed by hydrazinolysis of the phthalimide groups. The maximum amount of the amine-containing units in the resulting A-HBPGs can reach 31 mol%. The introduction of higher amounts of these units is limited by the reactivity (sterical) and viscosity factors. A-HBPGs are effective in CO_2_ capture from the ambient air. The measurements confirm that the amount of adsorbed carbon dioxide is proportional to the number of amine sites in the adsorbing polymer. It should be mentioned that such types of CO_2_ sorbents can be regenerated by heating, because desorption proceeds above 80 °C.

It is well known that many factors influence the successful application of CO_2_ sorbents in real-life systems. Generally, the properties of such sorbents are related to the materials used for their preparation, as well as the conditions during sorption and desorption processes. In addition, a good sorbent should be stable over many adsorption–desorption cycles. The results of our studies show that, in the case of CO_2_ adsorbents based on various supports and amine-containing hyperbranched polymers (polyamines and polyglycerols), the presence of steel coil and glass sphere adsorbents contributes to a rapid decrease in the CO_2_ capture abilities of the material. However, adsorbents consisting of A-HBPGs or polyethyleneimine (PEI) and fumed silica are capable of adsorbing significant amounts of CO_2_, up to 97.5 mg and 626 mg per gram, respectively. These values are equivalent to ca. 40–49% of nitrogen atoms present in the sample, being involved in the sorption process. The A-HBPG-containing sorbents are stable for at least 17 adsorption–desorption cycles, provided that the desorption is performed in an oxygen-free atmosphere [77].

**Figure 3:**
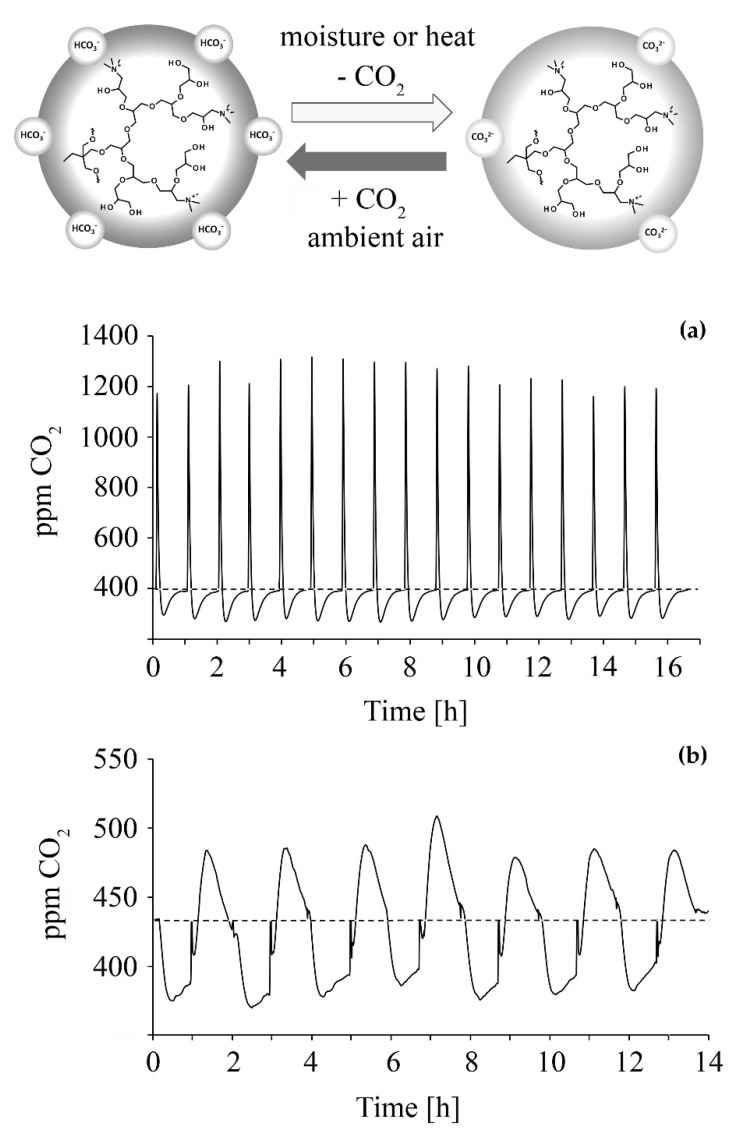
Hyperbranched polyethers with ammonium groups: (**a**) absorption–desorption curve for seventeen cycles, with temperature-induced desorption; (**b**) absorption–desorption curve for seven cycles of moisture change from 37% to 0% relative humidity.

Since the thermal desorption of CO_2_ may be expensive and not environmentally friendly, we developed systems based on hyperbranched polyglycerols containing trimethylammonium groups capable of the reversible capture/release of CO_2_ under humidity control (Figure 3) [78]. Usually, the full sorption capacity of such sorbents is achieved for a moderate relative humidity level (20–40%). In these conditions, they are capable of capturing (in the form of bicarbonate moieties) up to 42 mg of CO_2_ per gram, and approximately 20% (up to 8.2 mg per gram) of this amount could be reversibly desorbed and absorbed under various conditions (Figure 3). The typical size of the humidity or temperature swing is estimated to be in the range of 0.9−1.1 mg of CO_2_ per gram per hour. In the case of the humidity swing, the absorption and desorption times are on comparable levels, while, for thermal desorption, a short temperature impulse is only needed to fully regenerate the bed. The investigated sorbents are stable for several capture/release cycles and are promising materials for CO_2_ capture.

The sorbents that are susceptible to humidity swing—a mechanism that allows for the almost costless desorption of captured CO_2_ by increasing the humidity of the air, or to regeneration with short temperature impulses—are particularly interesting from an environmental and economic point of view [79,80,81]. However, polymeric systems based on these mechanisms are relatively rare in the literature. Research concerning increasing sorption capacity, chemical character, swing size, or long-term stability of these systems is still required [82,83].

## 7. Conclusions

Carbon dioxide is an ideal raw material for chemical synthesis, as it is a rather safe, cheap, renewable, and abundant compound. There are a number of processes in which CO_2_ can be used directly as a comonomer in chain polymerization processes or as one of the reactants involved in polymer synthesis by multi-step procedures [84]. They include traditional technologies where CO_2_ is employed in the synthesis of urea or formaldehyde, as well as the emerging ones based on reactions of CO_2_ with heterocyclic compounds.

In this contribution, we present a number of original solutions, the key elements of which are the synthesis of five-membered cyclic carbonates in the reaction of CO_2_ with oxiranes and their further ester exchange and/or step-growth polyaddition reactions with diols and diamines. New synthetic procedures leading to oligomeric carbonate diols, polycarbonates, and polyurethanes seem to be particularly attractive due to the possibility of eliminating toxic reagents, such as phosgene and diisocyanates. We have also shown that a functionalized five-membered cyclic carbonate can serve as a versatile precursor for a wide variety of hyperbranched polymers and networks.

Studies carried out at the CCTP on the physicochemical, mechanical, and biodegradable properties indicate that some of the CO_2_-based polymers can be suitable candidates for practical application as adhesives, biomaterials, smart materials, and components of CO_2_ capture systems. Finally, it is worth noting that the recently developed metal-based and organocatalytic systems can be effectively applied to the preparation of a large family of structurally and chemically complexed bio-based cyclic carbonates, which can be easily obtained from epoxidized vegetable oils, sugars, and residual agricultural biomasses [17,52,85,86,87]. Although synthetic strategies concerning the valorization of CO_2_ and biomass to valuable polymeric materials are still in their early stages, they may offer exciting possibilities to create a renewable carbon economy and contribute to the more sustainable use of resources in the coming years. It is anticipated that the development of more challenging technologies based on the reduction of CO_2_ under mild conditions, as well as the straightforward catalytic formation of C–C bonds, will lead to new prospects, such as the direct synthesis of ethylene glycol via the reductive coupling of CO_2_ or the synthesis of acrylic acid from ethylene and CO_2_ [88].

## Data Availability

Data sharing not applicable.
